# Combination Therapy With Adalimumab Plus Intensive Granulocyte and Monocyte Adsorptive Apheresis in Patients With Refractory Ulcerative Colitis

**DOI:** 10.14740/jocmr2333w

**Published:** 2015-09-25

**Authors:** Satoshi Tanida, Tsutomu Mizoshita, Hirotada Nishie, Keiji Ozeki, Takahito Katano, Eiji Kubota, Hiromi Kataoka, Takeshi Kamiya, Takashi Joh

**Affiliations:** a Department of Gastroenterology and Metabolism, Nagoya City University Graduate School of Medical Sciences, Nagoya City Aichi Prefecture, Japan

**Keywords:** Ulcerative colitis, Clinical remission, Mucosal healing, Long-term efficacy

## Abstract

**Background:**

The efficacy and safety of combination therapy with adalimumab (ADA) plus intensive granulocyte and monocyte adsorptive apheresis (GMA) (two sessions per week) for the treatment of refractory ulcerative colitis (UC) have not been previously evaluated.

**Methods:**

This retrospective study evaluated the 10-week efficacy of combination therapy with ADA plus intensive GMA on refractory UC patients, on clinical outcomes over 52 weeks under subsequent maintenance monotherapy of ADA, and the effect of combined azathioprine (AZA) with ADA at failure to achieve clinical remission at 10 weeks and at flare-up by 52 weeks. Ten patients were given initial combination therapy of ADA (160/80/40 mg every other week) plus intensive GMA. One patient received total colectomy because of poor response.

**Results:**

Of nine patients who received this combination therapy, 55.6% displayed cumulative clinical remission at 10 weeks and 33.3% displayed such remission at 52 weeks under subsequent maintenance monotherapy of ADA. The percentage of patients with mucosal healing at 10 weeks (endoscopy subscore ≤ 1) was 66.7%. Adverse events were observed in three patients (pneumonia, cerebral infarction and headache).

**Conclusion:**

It was concluded that combination therapy with ADA plus intensive GMA is useful for induction of clinical remission in refractory UC patients, and is well tolerated.

## Introduction

Ulcerative colitis (UC) is characterized by mucosal ulceration, rectal bleeding, diarrhea, and abdominal pain. Studies have demonstrated the efficacy and safety of adalimumab (ADA) for treatment of patients with moderate-to-severe UC who had failed to achieve clinical remission or to respond to conventional therapy consisting of corticosteroids, azathioprine (AZA), and/or aminosalicylic acid (5-ASA) [1]. However, the efficacy of ADA monotherapy for induction of clinical remission in randomized patients that had failed to respond to conventional therapy was only 16.5% after 8 weeks of therapy [1]. Granulocyte and monocyte adsorptive apheresis (GMA) with Adacolumn^®^ (JIMRO, Takasaki, Japan) is another effective and safe therapeutic option for patients with mild-to-moderate UC that is refractory to pharmacological therapy [2]. Furthermore, intensive GMA, involving two sessions per week, has recently been shown to be superior to routine weekly GMA both in terms of remission rate and time to remission in patients with refractory UC [3]. However, this approach is also considered to be limited because the efficacy of intensive GMA for induction of clinical remission in patients with severe UC is not satisfactory [4].

Here we report a retrospective assessment of the efficacy of 10 weeks of combination therapy with ADA plus intensive GMA for refractory UC patients, the clinical outcomes over 52 weeks under subsequent maintenance monotherapy of ADA, and the effects of combined AZA with ADA treatment at failure to achieve clinical remission at 10 weeks and at flare-up after 52 weeks.

## Patients and Methods

### Patients

Between July 2013 and July 2015, 10 consecutive patients with moderate and severe UC were recruited in Nagoya City University Hospital for this study. Patients receiving a combination therapy of ADA plus intensive GMA for corticosteroid-refractory or -dependent UC or for refractory UC that showed loss of response to tacrolimus (TAC) were enrolled. All study protocols were approved by the Nagoya City University ethics committee and written consent was obtained from all patients prior to enrollment.

### Treatment and assessments

The primary outcome was the clinical remission rate at 10 weeks of patients receiving a combination therapy of ADA plus intensive GMA, and the secondary outcomes were the clinical remission rate at 52 weeks under subsequent maintenance monotherapy of ADA, and the clinical course over 52 weeks under combined AZA with ADA treatment at failure to achieve clinical remission at 10 weeks and at flare-up by 52 weeks. Disease activities and severities were assessed using a full Mayo score [5] at baseline, 10 weeks, and 52 weeks, and a partial Mayo score at 24 weeks. Flare is defined as a combination of rectal bleeding with an increase in stool frequency as previously reported [6]. Patients were enrolled in this study according to the following criteria: 1) corticosteroid-refractory or corticosteroid-dependent UC; or 2) loss of response to TAC; and 3) patients with moderate-to-severe UC (Mayo score of 6 - 12 points at baseline) including an endoscopic subscore of 1 - 3 points despite concurrent treatment with corticosteroids, and/or TAC, and 5-aminosalicylates. Patients who failed to complete a 10-week combination therapy of ADA plus intensive GMA were excluded from clinical assessment as discontinued cases. Only corticosteroids dosages were tapered off as appropriate. Any adverse event, including date of onset, severity, and outcome, and the relationship of such events to these therapies were recorded.

### Statistical methods

The data are presented as means ± SE, and comparisons were made by using a paired *t*-test. A significance level of 0.05 was used for all statistical tests, and two-tailed tests were applied when appropriate. The cumulative clinical remission rate was also calculated using the Kaplan-Meyer method.

## Results

The demographic data of the study patients are shown in Table 1. The mean age was 57.1 years old, and the mean disease duration was 8.3 years. The involved colon portion included extensive colitis (eight patients) and left-sided colitis (two patients). Concurrent medications included 5-aminosalicylates, corticosteroids and TAC. Of the 10 patients, three were corticosteroid-refractory, and seven were corticosteroid-dependent, and two experienced loss of response to TAC. The mean full Mayo score and median C-reactive protein (CRP) levels at baseline were 9.3 points and 1.28 mg/dL, respectively. The mean doses of corticosteroids were 26.5 mg daily (Table 1).

**Table 1:** Baseline Demographic Variables of the 10 Cases With UC Refractory to Medications Including TAC Who Were Selected for This Combination Therapy With ADA Plus Intensive GMA

Demographic	Number of patients
Male/female	6/4
Age (range)	57.1 (34 - 85)
Mean disease duration, years (range)	8.3 (0.2 - 20)
Disease location (n, %)	
Extensive	8 (80)
Left-sided	2 (20)
Concurrent medication (n, %)	
5-ASA	10 (100)
Corticosteroids	10 (100)
TAC	2 (20)
Baseline full Mayo scores (mean)	9.3
Median CRP levels (range)	1.28 (0.03 - 27.9)
Doses of corticosteroids, mg/day (mean)	26.5
Corticosteroid-dependent/refractory (n, %)	
Dependent	7 (70)
Refractory	3 (30)

The data are presented as means or median values.

**Figure 1:**
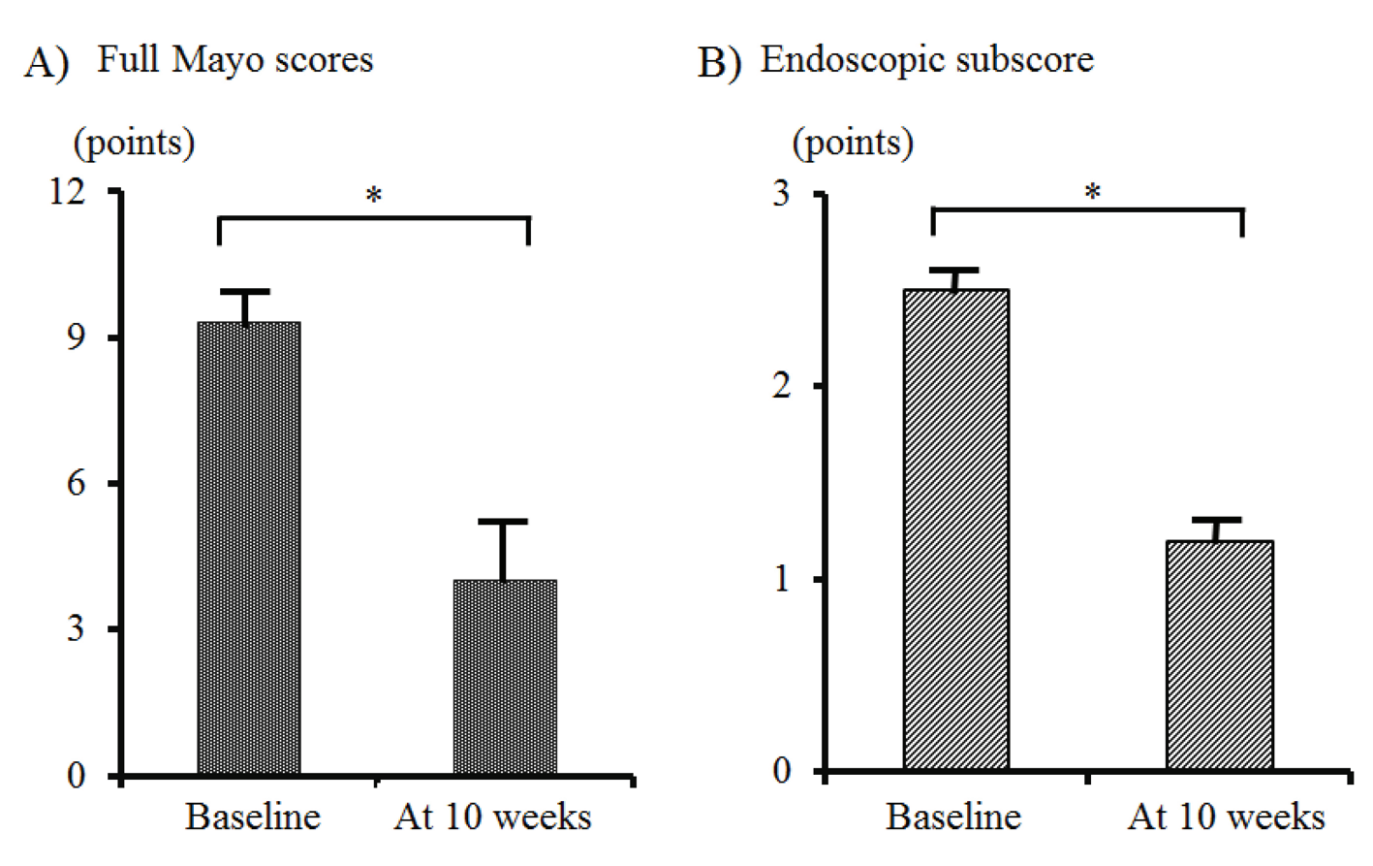
Changes in the mean full Mayo scores (A) and endoscopic subscore (B) between baseline and 10 weeks in nine cases. No. 4 patient, who received total colectomy, was excluded from this analysis. The data are presented as means ± SE values, and comparisons were made by using a paired *t*-test. A significance level of 0.01 was used for all statistic tests, and two-tailed tests were applied when appropriate.

All 10 patients were initially given a combination therapy of ADA (160/80/40 mg every other week) plus intensive GMA (two sessions per week). One case received total colectomy because of no response to this combination therapy. For the other nine cases, the mean full Mayo score and the endoscopic subscore changed from the baseline values of 9.0 ± 0.68 points and 2.4 ± 0.24 points, respectively, to 3.1 ± 0.8 points and 1.2 ± 0.22 points, respectively, at 10 weeks (Fig. 1A, B). These scores were improved with a significant difference (P < 0.01). The mean CRP level of nine patients that underwent combination therapy with ADA plus intensive GMA was 1.08 ± 0.30 mg/dL at baseline. By 10 weeks, this level had significantly decreased to 0.21 ± 0.07 mg/dL (P < 0.05) (Table 2).

**Table 2:** Clinical Course Over 52 Weeks

No.	Age (years)	Sex	Disease location	Pretreated		Baseline			Treated		At 10 weeks					At 24 weeks		At 52 weeks				Clinical course			
				TAC LOR	PSL (mg)	Full Mayo	Endo scores	CRP (mg/dL)	ADA	Intensive GMA	Full Mayo	Endo scores	CR	CRP (mg/dL)	PSL (mg)	p Mayo	CRP (mg/dL)	Full Mayo	Endo scores	CR	CRP (mg/dL)	ADA continued	Failed/flare (weeks)	AZA (mg)	AE
1	50	M	Left-sided	-	5	8	3	0.04	+	+	5	2	-	0.04	0	0	0.02	1	1	+	0.02	+	-	-	-
2	34	F	Left-sided	-	5	6	2	0.3	+	+	2	1	+	0.13	0	1	0.06	2	1	+	0.06	+	32	50	-
3	65	M	Extensive	+	40	11	3	2.52	+	+	2	1	+	0.34	0	1	0.14	2	1	+	0.1	+	-	-	-
4	71	F	Extensive	-	40	12	3	27.9	+	+	12 (4 weeks)		ope		40 (4 weeks)										-
5	43	M	Extensive	-	60	12	3	0.55	+	+	6	1	-	0.03	0	1	0.03	1	0	+	0.03	+	12	25	+
6	85	M	Extensive	-	60	9	2	1.7	+	+	1	1	+	1.1	0	0	0.09	1	1	+	0.25	+	-	-	+
7	52	M	Extensive	-	15	10	3	2.03	+	+	1	1	+	0.08	0	0	0.09	3	1	-	0.3	+	56	50	-
8	35	F	Extensive	-	10	6	1	0.03	+	+	0	0	+	0.03	0	0	0.03	1	1	+	0.03	+	-	-	-
9	77	F	Extensive	+	15	10	3	1.55	+	+	3	2	-	0.09	0	1	0.03	2	1	+	0.03	+	10	25	+
10	59	M	Extensive	-	15	9	2	1.0	+	+	7	2	-	0.12	10	2	0.03	3	1	-	0.03	+	10	25	-

One patient received total colectomy at 4 weeks because of no response to combination therapy of ADA plus intensive GMA. TAC: tacrolimus; LOR: loss of response; PSL: prednisolone; CRP: C-reactive protein; ADA: adalimumab; GMA: granulocyte and monocyte adsorptive apheresis; p Mayo: partial Mayo; CR: clinical remission; failed: failure to achieve clinical remission; AZA: azathioprine; AE: adverse events.

**Figure 2:**
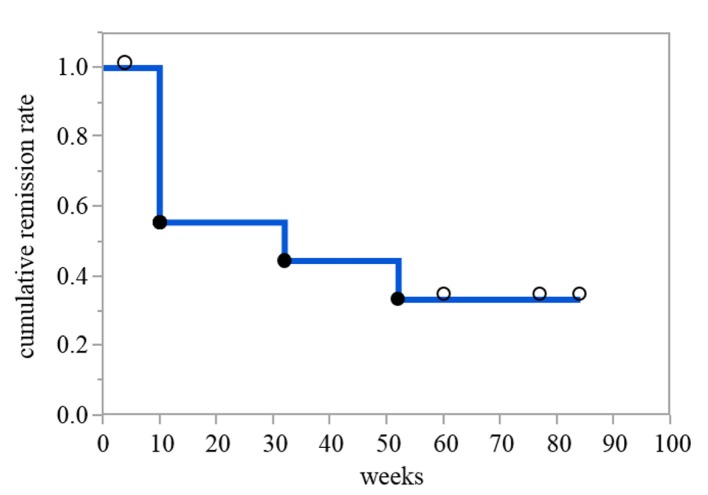
Cumulative remission rate of patients receiving combination therapy of ADA plus intensive GMA for 10 weeks and subsequent maintenance monotherapy of ADA. A Kaplan-Meier plot with a fitted curve of cumulative remission rate over time is shown. The solid circles represent event occurrences and censoring is indicated by the open circles.

Of the nine patients who received combination therapy with ADA plus intensive GMA, 55.6% showed cumulative clinical remission at 10 weeks, and 33.3% showed cumulative clinical remission at 52 weeks following subsequent maintenance monotherapy of ADA. The percentages of patients with mucosal healing (defined as endoscopy subscore ≤ 1) and complete mucosal healing (endoscopy subscore = 0) [7] at 10 weeks were 66.7% and 11.1%, respectively. Moreover, when AZA was combined with ADA for treatment of patients who failed to achieve clinical remission at 10 weeks or who experienced flare-up by 52 weeks, the percentages of clinical remission and clinical response at 52 weeks were 77.8 % and 100%, respectively. In addition, of the nine patients, corticosteroids could be withdrawn at 10 weeks in eight patients (88%) (Table 2, Fig. 2).

### Safety

Adverse events were observed in three patients: one case of pneumonia, one case of cerebral infarction without any persistent paralyses and one case of headache. There were no cases of lymphoma, non-melanoma skin cancer, leukemia, or tuberculosis in the present study. Combination therapies with ADA and intensive GMA or AZA were safe and well tolerated.

## Discussion

We have reported herein the efficacy of the combination of ADA induction administration with intensive GMA in 10 consecutive cases with refractory UC, and the 52-week efficacy of the combination of ADA maintenance administration with AZA for treatment of patients who failed to achieve clinical remission or who experienced flare-up by 52 weeks.

In clinical settings, ADA is an efficacious therapy for induction and maintenance of clinical remission in patients with moderately-to-severely active UC [7, 8]. A previous study of long-term remission and maintenance of UC with ADA (ULTRA2) showed that the overall percentage of patients showing clinical remission at week 8 was 16.5% in patients randomized to ADA and 9.3% in patients allocated to placebo (P = 0.019); the corresponding values for week 52 were 17.3% and 8.5%, respectively (P = 0.004). The percentage of anti-TNF-α naive patients that showed clinical remission at week 8 was 21.3% in patients randomized to ADA and 11% in patients allocated to placebo (P = 0.017); the corresponding values for week 52 were 22% and 12.4%, respectively (P = 0.029) [1]. In addition, the active ulcerative colitis trials 1 (ACT1), which evaluated the efficacy of infliximab (IFX) for induction and maintenance therapy in patients with UC, reported that the overall percentage of patients showing clinical remission at week 8 was 38.8% in patients randomized to 5 mg of IFX and 14.9% in patients allocated to placebo (P < 0.001); the corresponding values for week 54 were 34.7% and 16.5%, respectively (P = 0.001) [9]. Therapeutic treatment of ADA monotherapy for refractory UC is thus limited, which suggests that additional treatment is necessary for active UC patients who are biologically naive and show a loss of response to TAC. In the present study, 55.6% of the nine patients who received combination therapy with ADA plus intensive GMA showed cumulative clinical remission at 10 weeks, and 33.3% showed cumulative clinical remission at 52 weeks under subsequent maintenance monotherapy of ADA. Based on these outcomes, the addition of intensive GMA to ADA monotherapy appeared to be an effective combination therapy that induced a clinical remission.

GMA is available in Europe and in Japan for the treatment of patients with active IBD that may have become refractory to standard drug-based medication, including TNF-α blockers. GMA depletes elevated and activated myeloid lineage leucocytes and has been associated with a marked down-regulation of inflammatory cytokines including interleukin (IL)-1β, IL-6, IL-8, and TNF-α, which are released by myeloid leucocytes and lymphocytes, most likely via an upstream mechanism that involves adsorption of cytokine-producing cells [10, 11]. These findings suggest that combination therapy with ADA plus intensive GMA drastically down-regulates not only circulating inflammatory cytokines such as TNF-α due to GMA activity, but also dramatically down-regulates local TNF-α at microenvironmental sites in the gut mucosa due to ADA activity, thereby inducing a rapid clinical remission. In addition, serious adverse side effects have been rare in patients receiving GMA [4, 12].

In the present study, AZA was combined with ADA for treatment of patients who failed to achieve clinical remission at 10 weeks or who experienced flare-up by 52 weeks. The percentages of these treated patients showing clinical remission and clinical response at 52 weeks were 77.8% and 100%, respectively. A recent clinical trial demonstrated that anti-TNF-α naive patients with moderate-to-severe UC that were treated with IFX plus AZA were more likely to achieve corticosteroid-free remission at 16 weeks than those receiving either monotherapy. This conclusion was based on data that showed that 31 (39.7%) of 78 patients who received a combination of IFX and AZA achieved corticosteroid-free remission, compared with 17 (22.1%) of 77 patients who received IFX alone (P = 0.017) and 18 (23.7%) of 76 patients who received AZA alone (P = 0.032) [13]. However, serious adverse events were observed in 4% of the patients in the IFX/AZA combo group, and in 8% of the patients in the AZA group. In addition, regarding AZA safety concerns, there has been one trial that reported that one patient was dying of an infection associated with an immune-compromised state that occurred when taking AZA [14]. AZA/6-mercaptopurine (6-MP) is also associated with a 4 - 6 fold increased risk of lymphoma [15, 16] and a 2 - 6 fold increase in non-melanoma skin cancer [17, 18]. Thus, immunosuppressive therapy with AZA/6-MP is never without risk. Regarding safety, adverse events occurred in three cases over 52 weeks: one case of pneumonia, one case of cerebral infarction without any persistent paralyses and one case of headache. No serious adverse events were observed.

Based on these facts, combination therapy with IFX plus AZA is not always recommended from the start of treatment in patients with corticosteroid-refractory or -dependent UC, or in refractory UC patients who show loss of response to TAC. Starting with a combination therapy of ADA plus intensive GMA, it does not appear to be too late to delay treating with AZA combined with ADA until flare-up. Further prospective study is also needed to certify the efficacy of combination therapy of ADA with intensive GMA.

In conclusion, combination therapy with ADA plus intensive GMA is useful for induction of clinical remission in refractory UC patients, and is well tolerated.

## References

[1] Sandborn WJ, van Assche G, Reinisch W, Colombel JF, D'Haens G, Wolf DC, Kron M (2012). Adalimumab induces and maintains clinical remission in patients with moderate-to-severe ulcerative colitis. Gastroenterology.

[2] Ljung T, Thomsen OO, Vatn M, Karlen P, Karlsen LN, Tysk C, Nilsson SU (2007). Granulocyte, monocyte/macrophage apheresis for inflammatory bowel disease: the first 100 patients treated in Scandinavia. Scand J Gastroenterol.

[3] Sakuraba A, Motoya S, Watanabe K, Nishishita M, Kanke K, Matsui T, Suzuki Y (2009). An open-label prospective randomized multicenter study shows very rapid remission of ulcerative colitis by intensive granulocyte and monocyte adsorptive apheresis as compared with routine weekly treatment. Am J Gastroenterol.

[4] Hanai H, Watanabe F, Yamada M, Sato Y, Takeuchi K, Iida T, Tozawa K (2004). Adsorptive granulocyte and monocyte apheresis versus prednisolone in patients with corticosteroid-dependent moderately severe ulcerative colitis. Digestion.

[5] Schroeder KW, Tremaine WJ, Ilstrup DM (1987). Coated oral 5-aminosalicylic acid therapy for mildly to moderately active ulcerative colitis. A randomized study. N Engl J Med.

[6] Dignass A, Eliakim R, Magro F, Maaser C, Chowers Y, Geboes K, Mantzaris G (2012). Second European evidence-based consensus on the diagnosis and management of ulcerative colitis part 1: definitions and diagnosis. J Crohns Colitis.

[7] Reinisch W, Sandborn WJ, Panaccione R, Huang B, Pollack PF, Lazar A, Thakkar RB (2013). 52-week efficacy of adalimumab in patients with moderately to severely active ulcerative colitis who failed corticosteroids and/or immunosuppressants. Inflamm Bowel Dis.

[8] Reinisch W, Sandborn WJ, Hommes DW, D'Haens G, Hanauer S, Schreiber S, Panaccione R (2011). Adalimumab for induction of clinical remission in moderately to severely active ulcerative colitis: results of a randomised controlled trial. Gut.

[9] Rutgeerts P, Sandborn WJ, Feagan BG, Reinisch W, Olson A, Johanns J, Travers S (2005). Infliximab for induction and maintenance therapy for ulcerative colitis. N Engl J Med.

[10] Kashiwagi N, Hirata I, Kasukawa R (1998). A role for granulocyte and monocyte apheresis in the treatment of rheumatoid arthritis. Ther Apher.

[11] Saniabadi AR, Hanai H, Takeuchi K, Umemura K, Nakashima M, Adachi T, Shima C (2003). Adacolumn, an adsorptive carrier based granulocyte and monocyte apheresis device for the treatment of inflammatory and refractory diseases associated with leukocytes. Ther Apher Dial.

[12] Thanaraj S, Hamlin PJ, Ford AC (2010). Systematic review: granulocyte/monocyte adsorptive apheresis for ulcerative colitis. Aliment Pharmacol Ther.

[13] Panaccione R, Ghosh S, Middleton S, Marquez JR, Scott BB, Flint L, van Hoogstraten HJ (2014). Combination therapy with infliximab and azathioprine is superior to monotherapy with either agent in ulcerative colitis. Gastroenterology.

[14] O'Donoghue DP, Dawson AM, Powell-Tuck J, Bown RL, Lennard-Jones JE (1978). Double-blind withdrawal trial of azathioprine as maintenance treatment for Crohn's disease. Lancet.

[15] Armstrong RG, West J, Card TR (2010). Risk of cancer in inflammatory bowel disease treated with azathioprine: a UK population-based case-control study. Am J Gastroenterol.

[16] Beaugerie L, Brousse N, Bouvier AM, Colombel JF, Lemann M, Cosnes J, Hebuterne X (2009). Lymphoproliferative disorders in patients receiving thiopurines for inflammatory bowel disease: a prospective observational cohort study. Lancet.

[17] Long MD, Martin CF, Pipkin CA, Herfarth HH, Sandler RS, Kappelman MD (2012). Risk of melanoma and nonmelanoma skin cancer among patients with inflammatory bowel disease. Gastroenterology.

[18] Peyrin-Biroulet L, Khosrotehrani K, Carrat F, Bouvier AM, Chevaux JB, Simon T, Carbonnel F (2011). Increased risk for nonmelanoma skin cancers in patients who receive thiopurines for inflammatory bowel disease. Gastroenterology.

